# Adrenal Hemorrhage in Patients with Systemic Lupus Erythematosus and Antiphospholipid Syndrome: A Case Report and Literature Review

**DOI:** 10.1155/2023/6686168

**Published:** 2023-09-26

**Authors:** Weiwei Jiang, Danrui Chen, Daizhi Yang, Longyi Zeng, Wen Xu, Shuo Lin

**Affiliations:** ^1^Department of Endocrinology & Metabolism, The Third Affiliated Hospital of Sun Yat-Sen University, Guangzhou, Guangdong, China; ^2^Guangdong Provincial Key Laboratory of Diabetology, The Third Affiliated Hospital of Sun Yat-Sen University, Guangzhou, China; ^3^Guangzhou Municipal Key Laboratory of Mechanistic and Translational Obesity Research, The Third Affiliated Hospital of Sun Yat-Sen University, Guangzhou, China

## Abstract

Antiphospholipid syndrome (APS) is an autoimmune disorder while adrenal hemorrhage could be its rare complication. Herein, we report the case of a 32-year-old unmarried woman with a history of systemic lupus erythematosus (SLE) who was hospitalized after complaints of upper abdominal pain, limb weakness, and loss of appetite for 2 weeks. Laboratory examination revealed hyponatremia, low plasma cortisol levels, increased adrenocorticotropic hormone levels, and a positive anticardiolipin antibody status. Furthermore, computed tomography (CT) revealed the presence of bilateral adrenal masses. Ultimately, based on dynamic changes in CT images, these masses were diagnosed as adrenal hemorrhage owing to APS. A computer-assisted literature search was conducted to identify cases of primary adrenal insufficiency associated with APS and/or SLE. The clinical features, laboratory examination, treatments, and outcomes of these cases were summarized. Our findings emphasize the importance of screening for adrenal insufficiency in patients with SLE or APS who present with abdominal complaints, asthenia, and hyponatremia. It is also recommended to test for APS all patients with adrenal hemorrhage.

## 1. Introduction

Systemic lupus erythematosus (SLE) is an autoimmune disease that is characterized by damage to targeted tissues or organs owing to an aberrant immune response. The typical manifestations of SLE are malar rash, discoid lupus, and multiorgan disorders. The antiphospholipid antibodies are detected in the serum of approximately 20%–30% of patients with SLE, who tend to suffer from antiphospholipid syndrome (APS) during the flaring phase [1]. APS, a noninflammatory autoimmune disorder, is characterized by recurrent thrombogenesis, abortion, and a decreased platelet count. In individuals with APS, thrombogenesis can occur in any blood vessel, leading to ischemia and organ dysfunction [2]. Bilateral adrenal hemorrhage is the most common mechanism of Addison's disease in APS patients, but the possibility of an autoimmune origin of adrenal insufficiency should also be kept in mind [3]. Studies have reported that there are <1% of cases of APS-associated adrenal hemorrhage, with adverse outcomes such as adrenal insufficiency and adrenal crisis [4]. However, gastrointestinal complaints and musculoskeletal symptoms could be the only features of adrenal failure. Hyponatremia is the most common laboratory finding, reflecting mineralocorticoid deficiency. The main findings that led to the diagnosis of adrenal failure in newly diagnosed APS patients were hyponatremia and abdominal pain [3]. Adrenal hemorrhage secondary to APS is difficult to identify because of atypical symptoms such as abdominal pain, fever, altered mental status, and dynamic imaging presentations of the hematoma located in the adrenal glands.

Herein, we described the case of a 32-year-old Chinese woman with primary SLE who presented with adrenal insufficiency. After examinations, she was diagnosed with APS-associated adrenal hemorrhage. Furthermore, we reviewed existing literature on patients with SLE and/or APS and adrenal insufficiency.

## 2. Case Report

**Figure 1:**
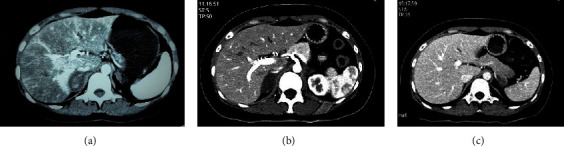
Enhanced bilateral adrenal CT: (a) bilateral adrenal masses with a significantly strengthened signal at the center of both the glands at onset; CT scan values on the left and right adrenal glands were 30 HU and 58 HU, respectively. (b) Two low-density nodular shadows located on both adrenals 4 days later. CT scan values on the left and right adrenal glands were 15 HU and 12 HU, respectively. (c) Atrophy of adrenal glands in the third-month follow-up. CT scan values on the left and right adrenal glands were 30 HU and 38 HU, respectively.

A 32-year-old unmarried woman with a history of SLE was admitted to a local hospital on October 27, 2021, owing to complaints of severe upper abdominal pain, limb weakness, and low appetite. She took hydroxychloroquine and prednisone for the treatment of SLE before and had discontinued the drugs for 1 year. She did not complain of nausea, vomiting, and fever. Her blood pressure, heart rate, and temperature were normal at the time of admission; however, serum sodium levels had decreased to 128 mmol/L. She was initially diagnosed with gastroenteritis. Although she received intravenous antibiotics, she continued to complain of upper abdominal pain and limb weakness. Thereafter, whole abdominal computed tomography (CT) revealed bilateral adrenal masses (see Figure 1(a) for the CT image taken at a local hospital). Spiral CT of these bilateral adrenal masses revealed that the center of the right mass was significantly strengthened, with a mean CT value of approximately 58 Hounsfield units (HU); the left mass had a CT value of 30 HU. Abnormal adrenocortical hormone levels were noted, with cortisol levels decreasing to 8.43 nmol/L (reference range, 185–624 nmol/L) at 8 AM and adrenocorticotropic hormone (ACTH) being >1250 pg/mL (reference range, 5–46 pg/mL) at 8 AM (refer to Table 1 for laboratory blood test results); therefore, the patient was diagnosed with adrenal insufficiency. The autoimmune workup confirmed the presence of lupus anticoagulant (LA) and anti-dsDNA, antihistone, anticardiolipin (aCL), and anti-*β*2 glycoprotein I antibodies (Table 2). The next day, her abdominal pain spontaneously disappeared; however, limb weakness and loss of appetite did not improve. Surgery was suggested by the doctor in local hospital to identify the nature of the bilateral adrenal masses. She refused to undergo surgery and was discharged with a prescription for hydrocortisone 20 mg at 8 AM and 10 mg at 4 PM.

On November 10, 2021, the patient was admitted to our hospital for further treatment. At the time of admission, she still complained of weight loss and limb weakness; however, she did not have a fever, abdominal pain, and hypotension (blood pressure, 111/89 mmHg). Physical examination revealed hyperpigmentation on her finger joints. However, no facial rash, oral ulceration, or Raynaud's phenomenon was observed. Meanwhile, her body and sexual organs had developed normally (height and weight were 155 cm and 50 kg, respectively). Examinations revealed hyponatremia, decreased plasma cortisol levels, and increased ACTH and renin levels. Also, the aldosterone level was normal (Tables 1 and 2). Furthermore, abnormal coagulation status was confirmed based on the significantly high activated partial thromboplastin time of 100.9 s (reference range 28.0–40.0 s). Meanwhile, erythrocytes, leucocytes, platelets, liver enzymes, creatinine, T-spot test, tumor and inflammatory makers, testosterone, estradiol, follicle-stimulating hormone, progesterone, luteinizing hormone, and 17-hydroxyprogesterone (17-OHP) were all within normal limits. A rheumatologist was consulted. Subsequently, abnormal autoimmune antibody and coagulation status findings confirmed the diagnosis of APS secondary to SLE [1]. She was administered hydroxychloroquine 100 mg two times a day and aspirin 0.1 g once a day. Furthermore, a prescription for hydrocortisone was provided to her as before. Moreover, instead of performing puncture surgery of the adrenal masses, repeated enhanced bilateral adrenal CT was performed, which revealed two low-density nodular shadows located on both adrenal glands; the right shadow was 30 × 20 mm and the left shadow was 20 × 15 mm (Figure 1(b)), with mean CT values of 15 and 12 HU, respectively. In other words, her adrenal glands were cystic rather than normal because the optimal critical value of the adrenal glands is 10 HU. Referring to ESE and ENSAT guidelines, if CT demonstrates a homogeneous adrenal mass with unenhanced HU between 11 and 20 and a tumor size <4 cm, an immediate additional imaging to avoid any follow-up imaging can be performed. The use of adrenal biopsy is recommended if clinical management would be altered by knowledge of the adrenal mass histology [5].

After carefully comparing these adrenal CT images, the patient was diagnosed with adrenal hemorrhage associated with APS secondary to SLE. Irregular antirheumatic therapy was considered a risk factor for adrenal hemorrhage for her. Nevertheless, weakness and anorexia gradually improved after hydrocortisone treatment during hospitalization. Finally, the patient was discharged with a prescription for hydrocortisone, hydroxychloroquine, and aspirin, as mentioned above.

During the third follow-up visit, on February 24, 2022, the patient was undergoing steroid replacement, antirheumatic, and aspirin therapy and remained in a good condition. Repeat CT scan values of the left and right adrenal glands were 30 and 38 HU, respectively (Figure 1(c)). However, laboratory tests still revealed decreasing serum cortisol and increasing ACTH levels at both 8 AM and 4 PM (Table 1). Considering hydrocortisone might not provide a sufficient suppression of the ACTH levels for its short-acting feature, the patient was prescribed prednisone 5 mg bid, hydroxychloroquine 100 mg bid, aspirin 0.1 g qd, and atorvastatin 20 mg qn. On March 21, 2022, she was confirmed to be 5 weeks pregnant. During her pregnancy, she stopped taking atorvastatin but continued taking prednisone, hydroxychloroquine, and aspirin without decreasing the dosage. Fortunately, in November 2022, she gave birth to a healthy baby girl without spontaneous abortion, thrombocytopenia, or recurrent thrombosis. She continued taking prednisone, hydroxychloroquine, and aspirin after delivery. However, she refused a reevaluation of adrenal function and imaging. Nevertheless, she required long-term follow-up.

## 3. Literature Review

We used the PubMed Central and EMBASE databases to perform a comprehensive Internet-based literature search. We used the keywords “systemic lupus erythematosus,” “antiphospholipid syndrome,” “adrenal insufficiency,” and “adrenal hemorrhage” and searched for articles published between 1980 and 2023. In total, 22 other cases of adrenal insufficiency owing to APS or SLE were identified [3, 6–19]. However, adrenal insufficiency has rarely been reported in patients with APS, and no common treatment guidelines are available.  Table 3 summarizes the clinical features, laboratory examination, treatments, and outcomes of these cases.

There were 13 (59%) men and 9 (41%) women. The mean age of the patients was 39.5 (28.75–49.75) years. Eight (36%) patients had connective tissue disease (CTD) before adrenal insufficiency, and five of these patients were diagnosed with SLE. Furthermore, in nine (41%) patients, adrenal insufficiency was the first manifestation of CTD. Four (18%) patients had hypothyroidism, with two of these patients having thyroiditis. Pulmonary embolism or deep venous thrombosis was observed in four (18%) patients. In one patient (patient 14), adrenal insufficiency was diagnosed 1 week after inguinal hernia surgery; on the other hand, in two patients, adrenal insufficiency was diagnosed after anticoagulant therapy was discontinued [6, 7].

Fever was observed in nine (41%) patients, hyperpigmentation in nine (41%), nausea or vomiting in seven (32%), abdominal pain in six (27%), fatigue or depression in five (23%), and weight loss in four (18%). Meanwhile, hyponatremia and hypotension were the key signs for doctors to diagnose adrenal insufficiency, which were observed in seven (32%) patients. Fifteen (68%) patients were positive for antinuclear antibody, and 11 (50%) patients were positive for LA. Of the 13 (59%) patients who were positive for aCL antibodies, seven had an isotype of aCL: three (43%) were positive for the IgG isotype, three (43%) for the IgM isotype, and one for both isotypes.

In most patients, CT or magnetic resonance imaging findings of the adrenal glands were different. Enlarged adrenal glands were observed in nine (41%) patients, adrenal atrophy in five (23%), and adrenal hemorrhage in six (27%), similar to adrenal masses. In three patients, adrenal gland images were normal.

As for the etiology of adrenal insufficiency, in many cases, owing to the unique and fragile vascular anatomy, adrenal hemorrhage secondary to a hypercoagulant state caused by SLE and/or APS was considered the reason, and patients who discontinued anticoagulants were high-risk groups. Eleven (50%) patients had a confirmed diagnosis of adrenal hemorrhage. Nevertheless, among these patients, autoimmune disorders of the adrenal glands could not be eliminated. In one study, a 21-hydroxylase antibody examination could not be conducted, and in another study, four patients were complicated with hypothyroidism [6, 8]. The latter finding is vital information for diagnosing the possibility of multiple endocrine organ dysfunction as a result of an autoimmune disorder.

All patients received steroid therapy, and hydrocortisone was the most commonly used medication (∼69%). Furthermore, 12 (55%) patients were administered anticoagulants. Patients undergoing long-term anticoagulant therapy remain well.

## 4. Discussion

Primary adrenal insufficiency is caused by the destruction of the adrenal cortex, leading to the insufficient secretion of glucocorticoids and mineralocorticoids. Here, we reported a case of adrenal insufficiency secondary to adrenal hemorrhage associated with APS that was confirmed via aPL testing and adrenal CT. SLE is a chronic autoimmune disease caused by the loss of self-tolerance and organ dysfunction. Bilateral adrenal hemorrhage is the most common mechanism of Addison disease in APS patients, but the possibility of an autoimmune origin of adrenal insufficiency should also be kept in mind [3]. However, when patients with SLE develop adrenal insufficiency with abnormally enlarged adrenal images, they cannot be diagnosed with adrenal hemorrhage owing to APS. The antiphospholipid antibody is an important diagnostic maker of APS and a risk factor for thrombosis and pregnancy complications [20]. The aPL test includes the evaluation of LA, aCL antibodies, and anti-*β*_2_ glycoprotein antibodies (IgG, IgM, and IgA). A positive LA test with or without a moderate-to-high titer of aCL or anti-*β*_2_GPI IgG or IgM is defined as a high-risk profile, providing additional confidence in the diagnosis [1].

Studies have reported that there are <1% of patients with APS who present with adrenal hemorrhage; therefore, it is a rare phenomenon during the course [4]. However, the main findings that led to the diagnosis of adrenal failure in newly diagnosed APS patients were hyponatremia and abdominal pain [3]. In particular, it is hard to identify this condition during the silent period. Nevertheless, continuous imaging may help in the diagnosis of this condition. The etiology of abnormally enlarged adrenal images in patients with adrenal insufficiency encompasses congenital adrenal hyperplasia (CAH), tuberculosis, autoimmune deficiency, tumor metastasis, infection, and hemorrhage [21]. On the other hand, patients with adrenal insufficiency generally have a chronic course because the clinical manifestations present only after there is ∼90% of adrenal cortex damage. However, bilateral adrenal hemorrhage or thrombosis may lead to acute adrenal insufficiency. During our literature review, we encountered a case of bilateral adrenal hemorrhage associated with APS that was reported by Bansal R et al. [9]. After carefully comparing the first adrenal CT image with the second, the evolvement of adrenal images was similar to the development of a hematoma. Considering the patient was diagnosed with APS and had an abnormal coagulation status, an APS-associated adrenal hemorrhage was identified.

At present, the pathogenesis of APS-associated adrenal hemorrhage remains unclear. One possible mechanism is associated with the unique vascular anatomy of the adrenal glands, making the organ vulnerable, particularly in the APS-induced hypercoagulable state. In bilateral adrenal, there are three arteries with tens of branches, and the transition from arteries to capillary is extremely rapid. Meanwhile, the single vein of the adrenal gland, comprising longitudinal muscle bundles, can hardly expand easily. Therefore, bilateral adrenal vein thrombosis may be the first pathological change during adrenal insufficiency secondary to APS, subsequently resulting in swollen adrenal glands. Finally, adrenal hemorrhage infarction is induced because of the occlusion of the vascular branches [20, 22]. The second possible mechanism is associated with the cellular characteristics of the zona fasciculata. The cells in this zone are rich in cholesterol owing to a high density of late endosomes. The membranes of these organelles contain lysophosphatidic acid, a target of antiphospholipid antibodies. These antiphospholipid bodies react locally and lead to cholesterol accumulation within the cell; this, in turn, leads to cell death and the release of lysosomal proteinases, activating endothelial cells, favoring coagulation, and causing microthrombosis [23].

The manifestations of APS-associated adrenal insufficiency are always typical, with abdominal pain, nausea or vomiting, hypotension, fever, and weakness being common symptoms. Most patients suffer from primary APS, and adrenal insufficiency may be the first manifestation of APS. Similar to most reported cases, our patient began complaining of abdominal pain, weakness, and anorexia. She was diagnosed with APS along with adrenal insufficiency. However, she did not have hypotension or fever. Furthermore, differential diagnosis was possibly difficult because the first CT scan values of the adrenal glands were not typical values indicative of adrenal hemorrhage. Given that imaging remains the most important tool in adrenal hemorrhage diagnosis, with CT scan being an important modality, the appearance of imaging varies with the age of the hematoma. Fresh hematomas usually demonstrate high attenuation which decreases over time. Magnetic resonance imaging in these cases can be useful in the differential diagnosis. Still, it cannot be available everywhere. In our case, it is possible that adrenal hemorrhage was the first manifestation of APS in our patient.

The treatment goals for APS include thrombogenesis prevention and pregnancy failure; furthermore, individual therapy is warranted. Therapeutic doses of low-molecular-weight heparin and subsequent vitamin K antagonists are first-line treatment options for first or recurrent APS-related venous thrombotic events (VTEs) [24]. According to European League Against Rheumatis recommendations, patients with APS and first unprovoked venous thrombosis should receive long-term treatment with vitamin K antagonists with a target international normalised ratio (INR) of 2-3. In patients with APS with first arterial thrombosis, treatment with VKA with INR 2-3 or INR 3-4 is recommended, considering the individual's bleeding/thrombosis risk [25]. On the other hand, glucocorticoid therapy is recommended for all patients with primary adrenal insufficiency. Guidelines for primary adrenal insufficiency suggest the use of hydrocortisone (15–25 mg) or cortisone acetate (20–35 mg) in two or three divided oral doses per day; the highest dose should be given in the morning at awakening [26, 27]. Nine patients had a history of thrombosis, and seven of them were treated with anticoagulants before the diagnosis of adrenal insufficiency [3, 6, 8–11]. In six (50%) of the twelve patients, heparin was the most commonly used anticoagulant. Among seven patients who received anticoagulants before adrenal insufficiency, the CT scan of only two patients revealed adrenal hemorrhage. Consistent with the guidelines, although heparin may cause adrenal hemorrhage, it should still be recommended to patients with APS-related VTEs owing to the extremely low incidence. However, based on the recommendations for the management of APS in China, we prescribed low-dose aspirin instead of heparin to our patient for primary prevention, complying with the primary prevention of APS-related VTEs [28]. During the follow-up, the patient was well without abortion or recurrent thrombotic events. Due to the potential—at least partial—recovery of adrenal insufficiency and regression of adrenal lesions, long-term reevaluation of adrenal function and imaging is necessary. However, due to the patient's own reasons, she refused to reexamine. Long-term follow-up should be necessary in the future.

In summary, primary adrenal insufficiency secondary to adrenal hemorrhage is a rare complication of SLE or APS. It is important to screen for adrenal insufficiency patients with SLE or APS presenting with symptoms of abdominal pain, weakness, and hyponatremia. On the other hand, it is also recommended to test for APS all patients with adrenal hemorrhage.

## Figures and Tables

**Table 1:** Laboratory data at the time of admission.

Item	Date			Reference range
	27 October 2021	08 November 2021	24 February 2022	
RBC (/L)	4.64	4.29	4.90	(3.8–5.1) × 10^12^
HGB (g/L)	123	116	141	115–150
WBC (/L)	5.96	6.45	6.56	(3.5–9.5) × 10^9^
PLT (/L)	286	229	387	(100–300) × 10^9^
K (mmol/L)	4.95	4.52	4.17	3.50–5.30
Na (mmol/L)	128	131	139	137–147
CL (mmol/L)	92	96.5	100.7	99.0–110.0
AST (U/L)	60.7	44	18	13–35
ALT (U/L)	29.8	30	9	3–35
GGT (U/L)	23.8	19	12	7–45
Urea (mmol/L)	4.00	5.38	3.36	2.4–8.2
CREAT (umol/L)	96	83	52	31.8–93.7
17-*α*-OPH (ng/mL)	—	0.19	—	0.09–4.00
24 h urinary free cortisol (nmol/24 h)	28.29	659.57	248.00	160–1112
24 h urinary K (mmol/24 h)	29.4	40.3	—	25.0–100.0
PT (s)	13.0	12.5	13.0	11.0–14.5
aPTT (s)	100.9	95.7	84.1	28.0–40.0
Cortisol rhythm (nmol/L)				
0 AM	9.59	—	43.53	185–624
8 AM	8.43	40.46	13.80	185–624
4 PM	7.63	—	78.17	<276
ACTH rhythm (pg/mL)				
0 AM	859	—	104	5–46
8 AM	>1250	—	>278	5–46
4 PM	1139	—	207	5–46
Renin (ng/L)	—	55.85	62.61	Recumbent position: 4–24 Upright position: 4–38
AARR	—	0.126	0.116	5.7
Angiotensin II (pg/mL)	—	80.67	101.79	Recumbent position: 25–129 Upright position: 49–252
Aldosterone (ng/dL)	—	7.03	7.26	Recumbent position: 1–16 Upright position: 4–31
Androstenedione (nmol/L)	—	<1.05	<1.05	1.0–11.5
DHEA–S (umol/L)	—	<0.41	<0.41	0.95–11.67
Lactogenic hormone (mIU/L)		692.1	1298.91	108.8–557.1
ds-DNA (IU/mL)	21.8	—	767	<30.0
ANA	1 : 1000	—	1 : 1280	<1 : 100

ANA = antinuclear antibody; aPTT = activated partial thromboplastin time; 17*α*-OPH = 17-alpha hydroxyprogesterone; ds-DNA = anti-double-strand DNA antibody; DHEA–S = dehydroepiandrosterone sulfate; Fib = fibrinogen; PT = prothrombin time.

**Table 2:** Antiphospholipid-antibody testing.

Item	Value	Reference range
aCL (IgG) (cu)	116	<12
Anti-*β*_2_ GPI (AU/mL)	134	<24
LA dRVVTs	3.12	<1.2
LA dRVVTc	1.27	
LA dRVVT NR	2.46	<1.2

aCL = anticardiolipin antibody; anti-*β*2GPI = anti-beta2 glycoprotein I antibody; dRVVT = dilute Russell viper venom time; dRVVTs = dRVVT screen; dRVVTc = dRVVT confirm; LA = lupus anticoagulant.

**Table 3:** Summary of the clinical features, diagnosis data, treatment, and etiology of all cases.

Case	Age (years)	Sex	Symptoms and signs	Underlying CTD	Serum cortisol (nmol/L) (7–9 AM: 145.4–619.6; 3–5 PM: 94.9–462.4)	ACTH (pg/ml) (5–50)	Immunological characteristics	Image investigation	Treatment	Etiology of Addison's disease
1 [3]	27	Male	Left hemithorax, fever; dyspnea; Raynaud's phenomenon; pulmonary embolism	Positive for Raynaud's phenomenon	Basal: 93 at 30 min: 88.3	483	ANA (+) acL (+); anti-*β*_2_GPI (+); LA normalised ratio (+)	Atrophy of the adrenal glands	Intravenous hydrocortisone 300 mg qd; intravenous hydrocortisone 50 mg qd; oral hydrocortisone 20 mg qd. low-molecular-weight heparin; coumarin anticoagulant	An acute microthrombotic mechanism or an acute microangiopathic mechanism of adrenal damage

2 [3]	54	Male	Fever; severe pain in the throat; difficulty in swallowing; deep vein thromboses; hyperpigmentation of the skin	APS	Basal: 320 at 30 min: 287	605	ANA (+) acL (+); anti-*β*_2_GPI (+)	Bilateral adrenal atrophy	Coumarin; hydrocortisone	Microangiopathic adrenal damage, but cannot completely eliminate the possibility of autoimmune destruction of the adrenal glands

3 [10]	44	Female	Fever; hemoptysis; debilitate, hypotension, depression; abdominal pain, nausea and vomiting; deep vein thromboses; hyperpigmentation	APS + SLE	190	21000	ANA (+); dsDNA (+) aCL (+); LA test (+)	The adrenal glands were normal on initial imaging, but later developed a diffusely thickened right adrenal gland and a 2.8 cm cystic mass in the left gland, which indicated involution of both the adrenal glands finally.	Hydrocortisone; subcutaneous heparin	Thrombosis of the adrenal vein and subsequent hemorrhagic necrosis of the adrenal glands

4 [11]	11	Male	Fever; dyspnea; cough; pulmonary embolus; anorexia; weight loss; vomiting	APS	74.52	NA	ANA (+) LA (+)	Punctate calcifications in both enlarged adrenal glands	Intravenous heparin and anticoagulation with coumadin; prednisone and fludrocortisone	Thrombotic events leading to acute adrenal involvement
5 [6]	43	Male	Right flank pain; nausea; vomiting; dizziness, hyponatremia; hypotension; deep vein thrombosis; pulmonary embolism	SLE + APS	Basal: 14 at 30 min: 320	NA	ANA (+); dsDNA (+) acL (+); anti-*β*_2_GPI (+); LA (+)	Bilateral bulky heterogeneous adrenal glands surrounded by fat	Intravenous hydrocortisone 100 mg tid; oral fludrocortisone and hydrocortisone; aspirin 81 mg qd; heparin; warfarin 2 mg po qd; hydroxychloroquine 300 mg po qd; mofetil 1000 mg po bid	Caused by APS, adrenal thrombosis led to hemorrhage after warfarin cessation

6 [12]	45	Male	Fatigue; loss of appetite; nausea; vomiting; hypotension; hyponatremia	SLE + APS	8 AM: 23	558.7	ANA (+); dsDNA (+) aCL (+); LA (+)	Hyperdense lesions in bilaterally enlarged adrenal glands, which finally shrunk	Intravenous methylprednisolone 48 mg qd; oral prednisone 55 mg qd, 30 mg qd, and 10 mg qd; heparin infusion 5000iu qd; warfarin; cyclophosphamide 50 mg qod; hydroxychloroquine 200 mg bid; oxcarbazepine 0.6g bid; entecavir 500 ug qd	Adrenal hemorrhage

7 [7]	50	Female	Abdominal pain; nausea; vomiting; mucosa hyperpigmentation; hypotension; hyponatremia; weight loss; pulmonary infection; left lower extremity swelling and pain; cough	SLE + APS	8 AM: 17	8 AM: 91	ANA (+); anti-SSA (+); dsDNA (+) aCL (+)	Slightly thick right external adrenal branch and left adrenal nodule	Hydrocortisone 40 mg daily at 8 : 00 PM and 20 mg daily at 4 : 00 PM; warfarin 2.5 mg qd	Thrombosis of the adrenal, but cannot eliminate the possibility of autoimmune disease
					4 PM: 0.88	4 PM: 5.5				
8–13 [8]	38.5 (28,49)	3 females and 3 males	Fever; hyperpigmentation; loss of appetite; depression; Raynaud's phenomena; anemia; deep vein thrombosis	33% SLE; 17% SLE + SS; 17% AS + APS; 17% Takayasu arteritis; 17% SSc	Serum cortisol level of all patients had decreased significantly	One SLE female patient had kept normal serum ACTH, while others increased	50% ANA (+), anti-SSA (+); 33% anti-dsDNA (+), RNP (+) 33% LA (+); 17% aCL (+)	Three patients showed normal images, one patient had adrenal gland hemorrhage, one had left adrenal nodule, and one was identified to have bilaterally enlarged adrenal glands with low-density nodule	Glucocorticoid; cyclophosphamide or sulfasalazine; hydrocortisone tablets 20–40 mg qd; rivaroxaban and intravenous heparin.	Thrombosis events or autoimmune destruction of the adrenal glands

14 [13]	51	Male	Fever; left-sided thoracic pain; hyponatremia; transient paranoid psychosis	Primary APS	>20	440000–540000	ANA (+) aCL (+); LA (+)	Enlargement of the right adrenal gland; slight decrease in the size of both the adrenals after 2 months, which further shrunk after 5 months	Intravenous hydrocortisone; prednisolone 25 mg qd; cortisone acetate 12.5 mg tid; fludrocortisone 0.05 mg qd; parenteral and oral neuroleptics; oral benzodiazepines	Bilateral adrenal hemorrhage, possibly hemorrhagic infarctions

15 [14]	24	Female	Intermittent headache; lower abdominal pain; vomiting; pale, icterus and mild splenomegaly	SLE	50	NA	ANA (+); anti-dsDNA (+)NA	Diffusely enlarged bilateral adrenal gland suggestive of bilateral adrenal hemorrhage	Oral steroids	Bilateral adrenal hemorrhage

16 [9]	50	Male	Deep venous thrombosis; fever; pulmonary embolism; hypotension and bilateral flank pain	APS	91	319	ANA (+); anti-SSA (+); anti-dsDNA (+) aCL (+)	Bilateral adrenal hemorrhage	Hydrocortisone; warfarin	Bilateral adrenal hemorrhage
17 [15]	63	Male	Right flank pain; fever, nausea; fatigue; hypotension; increasing pulse rate; mental confusion; skin hyperpigmentation of sun-exposed areas	APS	133	53	ANA (+) aCL (+); anti-*β*_2_GPI(+); LA (+)	Bilateral adrenal hemorrhage	Hydrocortisone; warfarin	Bilateral adrenal hemorrhage

18 [16]	45	Male	With a left lower extremity deep vein thrombosis and pulmonary embolism, bilateral flank pain	APS	Morning: 38.7	149	anti-*β*_2_GPI(+); aCL (+)	Bilateral adrenal gland edema	Hydrocortisone 30 mg qd	Adrenal hemorrhage

19 [17]	51	Female	Abdominal pain; unremitting nausea, vomiting, and watery diarrhea;	Catastrophic APS	—	—	ANA (+); aCL (+); anti-*β*_2_GPI (+);	Bilateral adrenal hemorrhage; ileus; bowel perforation	High-dose steroids, plasma exchange; anticoagulation	Bilateral adrenal hemorrhage

20 [18]	70s	Female	Fracture of the 2nd–7th ribs on the left with resultant hemothorax; abdominal pain	APS	—	—	aCL (+); anti-*β*2GPI(+); LA (+)	A recurrent hemorrhage of the left adrenal gland, a new hemorrhage of the right adrenal gland and two new hepatic lacerations	Warfarin; oral hydrocortisone and fludrocortisone	Bilateral adrenal hemorrhage

21 [19]	71	Female	Nausea and vomiting after the surgical treatment of a femoral fracture	—	4.95	275	—	An increased adrenal volume; 2 months later a reduction in the volume of both adrenal glands	Enoxaparin 4000 IU qd; cortisone acetate 37.5 mg qd; progressively cortisone acetate reduced to 12.5 mg qd	Heparin-induced thrombocytopenia likely is a cause, leading to adrenal vein thrombosis and adrenal hemorrhage.
22 [19]	58	Male	Pain, fever; intense asthenia	—	12.9	115.6	—	The imbibition of fat tissue around the left adrenal gland; 10 days later a marked increase in the volume of both adrenal glands; repeated imaging showed a progressive reduction in the volume of both adrenal glands	Enoxaparin 4000 IU qd; intramuscular hydrocortisone and then with cortisone acetate 75 mg qd; cortisone acetate 37.5 mg qd; cortisone acetate 12.5 mg qd	Bilateral adrenal apoplexy

aCL = anticardiolipin antibody; anti-*β*_2_GPI = anti-beta2 glycoprotein I antibody; ANA = antinuclear antibody; SS = Sjogren syndrome; AS = ankylosing spondylitis; SSc = systemic sclerosis; anti-dsDNA = anti-double-strand DNA antibody; anti-RNP = antiribonucleoprotein antibody; CTD = connective tissue disease; LA = lupus antibody; anti-SSA = anti-Ro/SSA antibodies; NA = not available.
